# Forced Eruption: Alternative Treatment Approach to Restore Teeth with Subgingival Structure

**DOI:** 10.1155/2022/9521915

**Published:** 2022-09-01

**Authors:** Hamid Kermanshah, Elmira Najafrad, Sara Valizadeh

**Affiliations:** ^1^Dental Research Center, Dentistry Research Institute, Tehran University of Medical Sciences, Tehran, Iran; ^2^Restorative Dentistry Department, School of Dentistry, Tehran University of Medical Sciences, Tehran, Iran; ^3^Oral Biological and Medical Sciences Department, University of British Columbia, Faculty of Dentistry, Vancouver, BC, Canada

## Abstract

The management of teeth with deep caries, fracture, or perforation in the cervical third of the root is an integral part of dental practice. Orthodontic extrusion preserves the natural root system and may convert the tooth indicated for extraction into useful tooth with good prognosis and a low risk of failure. Orthodontic tooth eruption can be an alternative to treatment such as surgical crown lengthening, especially in esthetic areas, and provides more favorable conditions for prosthodontic coronal restorations by guaranteeing proper sealing and esthetics and preserving periodontal tissue health. The aim of this case report was to explain a multidisciplinary approach that successfully preserve and treat the teeth with subgingival carious lesion. This innovative method is cost-effective and can be easily done with the equipment available in any office.

## 1. Introduction

Dentistry is currently making significant strides in meeting the ever-increasing dental and esthetic demands of the community and patients [1, 2]. This has challenged the deep-rooted treatment approaches and values of dentistry, including preserving natural teeth and the root system as long as clinically possible [3].

Esthetic advances in implantology pave the way for successfully and predictably replacing missing teeth, contributing to a “rush-to-implant “mentality, to the detriment of the self-preservation value for patients and the profession [4].

Studies have shown that in 90% of cases, the maxillary anterior facial bone wall thickness is <1 mm, and in 50%, it is <0.5 mm [5, 6]. As a result, thin facial bone walls mainly composed of bundle bone are susceptible to resorption after tooth extraction. Therefore, caution should be exercised when implants are placed in thin gingival biotypes, in which the post-extraction bone is prone to resorb at a high rate, especially in the esthetic zone [7].

In fractured or severely decayed teeth, especially in teeth with previous endodontic treatment, many dentists recommend that a severely damaged/broken endodontically treated tooth should be replaced by an implant [8].

In many cases, dentistry falls short of restoring the normal periodontal architecture, despite novel technologies and biomaterials, soft and hard tissue grafting techniques, and advances in implantology. These shortcomings give rise to sub-optimal esthetic outcomes, including loss of interdental papillae or marginal gingival heights not aligned esthetically with adjacent teeth [1, 9].

Orthodontic tooth extrusion has been advocated instead of sacrificing the natural root system [2, 7]. If this technique is applied correctly, it gives rise to the preservation of the natural root system and the associated periodontal structure and architecture, and the patient can enjoy years of additional service. It can also preserve adjacent supporting tooth structures and the choice for reconstruction with implants [9, 10].

Surgical extrusion is an alternative to extraction for teeth with crown-root fractures, cervical root fractures, and subgingival caries. It is based on the concept of relocating the affected area of a tooth to a supragingival position, leaving sound tooth structure exposed to improve tooth restorability, and providing space for the reestablishment of the biological width. It is widely accepted that the success of surgical extrusion mainly depends on an atraumatic extraction method with as little damage as possible to the cementoblast layer on the root surface; otherwise, progressive root resorption or ankylosis and marginal bone loss or tooth mobility will occur.

Orthodontic root extrusion was first introduced by Heithersay and Ingber [11] to preserve the biologic width, expose sound tooth structure for optimal placement of restorative margins, and achieve esthetics [12].

Orthodontic extrusion is not possible in these situations: unfavorable axial tooth position, compromised periodontal health, short roots that lead to inadequate crown-to-root ratio, and wide internal root form [13].

Orthodontic tooth eruption is the preferred treatment modality to avoid the negative consequences of surgical crown lengthening, especially in esthetic areas. It is an interdisciplinary treatment requiring the expertise of endodontists, periodontists, orthodontists, and restorative dentists [14].

Some of the advantages of orthodontic forced eruption are improved bone level, low cost, and less time; however, poor esthetic outcomes during treatment and the need for more patient cooperation are the disadvantages of this technique [15].

This paper describes two cases with orthodontic extrusion of one and four teeth and the multidisciplinary management of such teeth with subgingival margins.

## 2. Case Reports

### 2.1. Case 1

A 48-year-old female patient whose chief complaint was the replacement and esthetic appearance of her upper right lateral incisor, canine, and premolar teeth was referred for treatment to the Restorative Department, Faculty of Dentistry, Tehran University of Medical Sciences, Iran.

**Figure 1:**
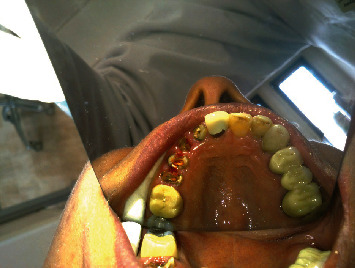
Occlusal view of central, lateral, canine, and premolars.

**Figure 2:**
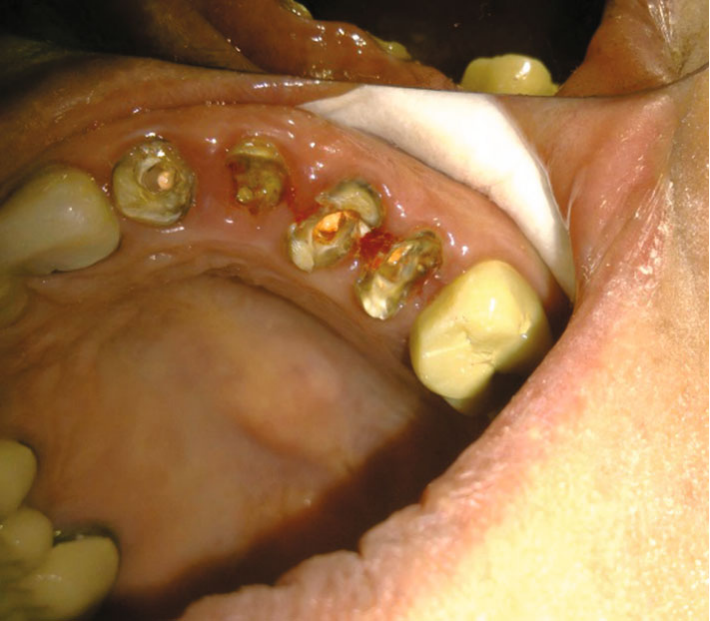
Occlusal and palatal view of central, lateral, canine, and premolars.

The teeth had been endodontically treated one month earlier. The patient's medical history was noncontributory. Clinical examination revealed an extensively damaged crown with thin mesial and palatal walls in the lateral incisor, a too short labial wall in canine, buccal and palatal walls in the first premolar, and palatal wall in the second premolar. The rest of the tooth structures were located 2–3 mm below the gingival margin (Figures 1 and 2).

**Figure 3:**
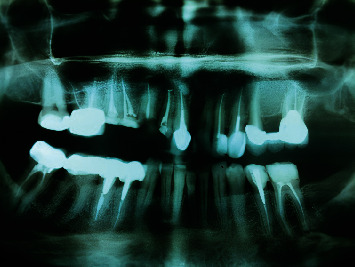
Panoramic radiographic view of patient's teeth.

**Figure 4:**
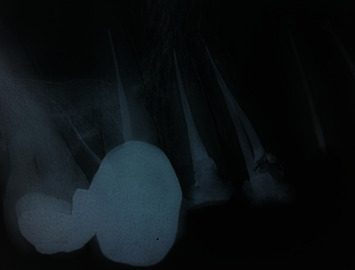
Periapical radiographic view of patient's teeth.

There was no tenderness on percussion or palpation. The periodontal condition of the teeth was normal with no pockets. The teeth had mobility within the normal limits (Grade 1) without any noticeable swelling. The radiographic examination confirmed that the roots had been endodontically treated without pathosis (Figures 3 and 4).

After analyzing factors such as the height of the smile line, patient's age, root anatomy, and financial resources, with the patient's consent, the teeth were decided to be treated through extrusion to allow the fabrication of crowns for these teeth to achieve improved esthetics and adequate biological width.

The estimated crown-root ratio and the remaining tooth structure were considered. Then, extrusion of approximately 4 mm for canine and 3 mm for the rest of the teeth was deemed adequate for achieving sufficient biologic width and a ferrule for the final restoration.

A fixed appliance was used in this case. After providing oral hygiene instructions (OHI) and implementing prophylactic measures, the carious lesions were eliminated. Because of the inadequate tooth structure remaining in the canine, approximately 5 mm of gutta-percha was removed from the root canal. A hook was fabricated with a piece of SS round wire (Dentarum, Inspringen, Germany), measuring 1 mm in diameter, with several artificial notches on its body for improved retention following cementation. The hook was cemented in the canine root canal with zinc phosphate cement (Harvard, Dahlwitz-Hoppegarten, Germany). For the rest of the teeth, after composite resin build-up, a composite resin bottom was bonded at the gingival area of their remaining buccal wall.

**Figure 5:**
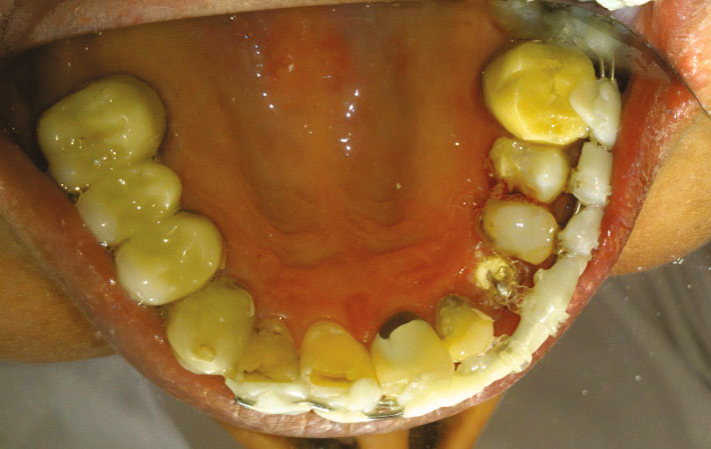
Occlusal view of fixed appliance used for orthodontic extrusion of four teeth.

**Figure 6:**
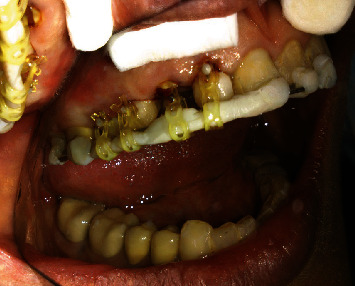
Buccal view of fixed appliance used for orthodontic extrusion of four teeth.

Also, an archwire was shaped conforming to the upper arch and fixed on the buccal surface of the teeth, extending from the first upper right molar to the upper left lateral incisor and covered by flowable composite resin. The wire was adjusted so that it did not interfere with protrusive and excursive movements. An elastic thread was passed between the hook and the archwire. The distance between the hooks and wire was determined, and the elastics were connected to the hooks on the provisional crown, curling around the supporting wire (Figures 5 and 6). The elastic thread was replaced with a shorter one every seven days until predetermined extrusion was achieved. The patient was instructed on how to use fresh elastics daily and to wear them continuously.

**Figure 7:**
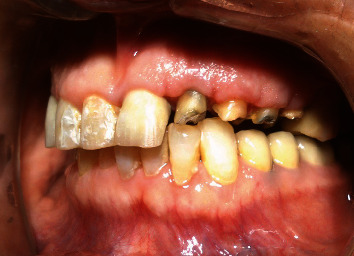
Buccal view of force erupted teeth after crown lengthening.

**Figure 8:**
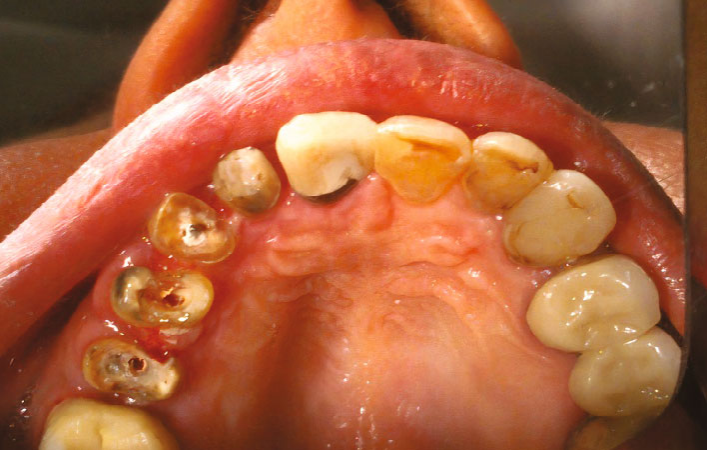
Occlusal view of force erupted teeth after crown lengthening.

Periodic evaluations were carried out every two weeks, and movements were measured for two months. After achieving favorable extrusion, the supporting wire and composite resin were removed, followed by a crown-lengthening procedure and circumferential fiberotomy to rearrange gingival, bone, and periodontal fiber levels to conform to the tooth new position (Figures 7 and 8). No active force was applied during a 60-day stabilization period. The extruded tooth was splinted to the adjacent teeth using composite resin to maintain the tooth position achieved by extrusion.

**Figure 9:**
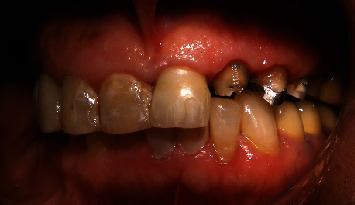
Buccal view of core preparation.

**Figure 10:**
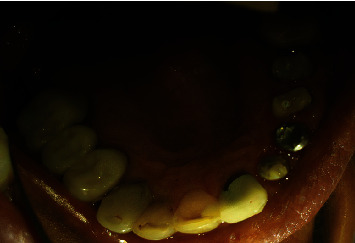
Occlusal view of core preparation.

After a two-month maintenance period, the wire was removed, and the interim crown and hooks were trimmed with diamond burs. PFM crowns were fabricated for these four teeth after core preparation (Figures 9 and 10).

The patient was followed periodically for six years. No signs of postoperative gingival inflammation and other periodontal changes were detected.

### 2.2. Case 2

**Figure 11:**
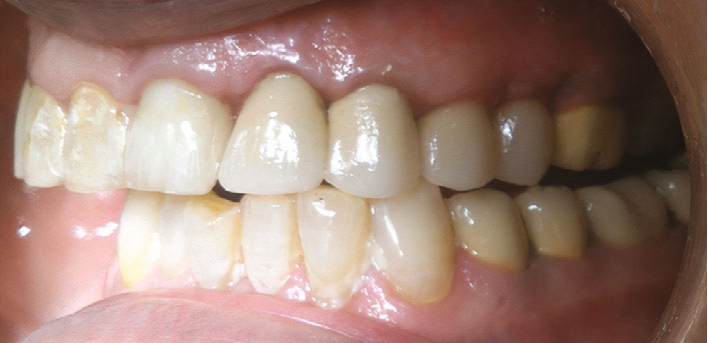
Buccal view of PFM crowns for upper left lateral, canine, and premolars.

**Figure 12:**
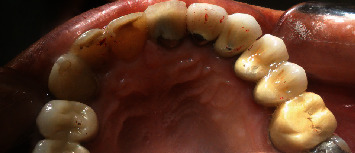
Occlusal view of PFM crowns for upper left lateral, canine, and premolars.

**Figure 13:**
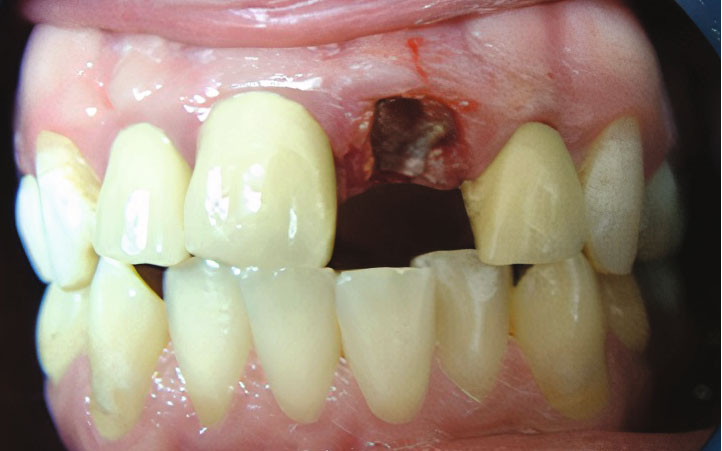
Frontal view of incisor teeth.

A 32-year-old male patient was referred to the Restorative Dentistry Department, Faculty of Dentistry, Tehran University of Medical Sciences, Iran, with the chief complaint of a dislodged crown of his upper left central incisor three times. The patient also wanted to change the PFM crowns of maxillary incisors. He reported no other associated symptoms (Figures 11 and 12) The patient's medical and familial histories were noncontributory, and the extraoral examination results were unremarkable. Clinical examination showed that the tooth structure in the left central incisor was located 2–3 mm below the gingival margin due to extensive decay, with inadequate ferrule to support the crown. Therefore, crown dislodgment had happened many times (Figure 13).

**Figure 14:**
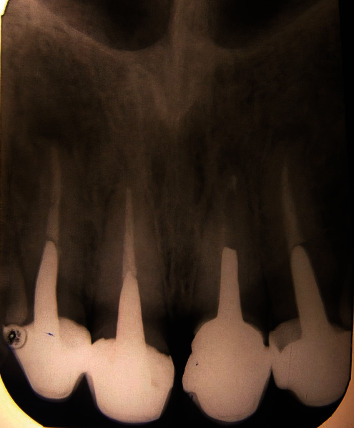
Radiographic view of patient's teeth.

The teeth were asymptomatic and responded negatively to palpation and percussion. There was adequate keratinized gingiva around the tooth, with no inflammation, and the gingival biotype was thin. Periodontal probing depths around the tooth were within the normal range. Radiographic examination showed that the patient's all maxillary incisors were root canal-treated, with custom metal posts and cores; excessive removal of gutta-percha from root canal was noticed in the left central incisor (Figure 14).

A consent form was obtained from the patient for the treatment plan, which consisted of a multidisciplinary approach for plaque control and oral hygiene instructions, orthodontic extrusion and conservative endodontic retreatment of the upper left central incisor, periodontal surgery, and restoration of maxillary incisors.

**Figure 15:**
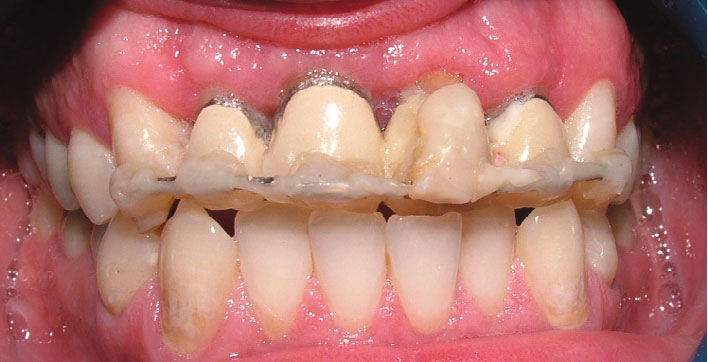
Extrusion with 19-gauge stainless steel arch wire.

By considering the estimated crown-to-root ratio and the carious tooth structure in the upper left central incisor, 3–4 mm of extrusion was deemed adequate for achieving sufficient biologic width and ferrule for the final restoration. After removing the carious lesions from the tooth, the previously dislodged post-and-core crown was used to ensure that the anterior region would remain esthetically pleasing during orthodontic forced eruption treatment, with no need to make a temporary crown. The crown was cemented with zinc polycarboxylate cement (Harvard, Dahlwitz-Hoppegarten, Germany); a dentinal pin was then bonded to the most cervical part of the buccal surface of the crown perpendicularly, using composite resin restorative material (Z250: 3 M ESPE, St. Paul, MN, USA) to create a hook for elastic insertion. The wire was made to conform to the upper arch shape using a round 19-gauge rigid stainless steel and bonded to the buccal surface of adjacent teeth (the right canine to the left canine) using the composite material. An elastic thread was passed between the pin and the wire (Figure 15).

**Figure 16:**
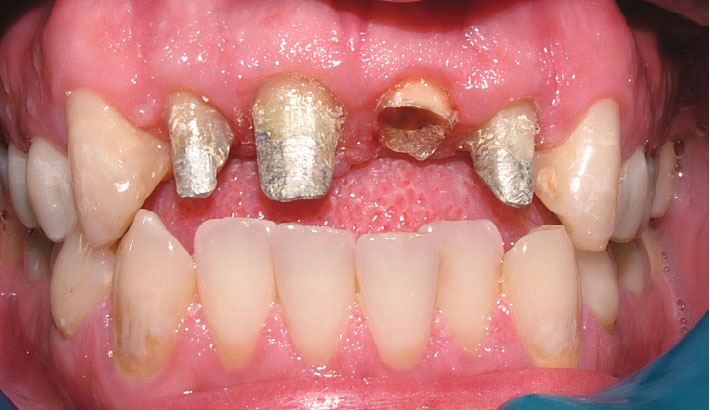
After extrusion and remove of other incisors crowns elastic force.

**Figure 17:**
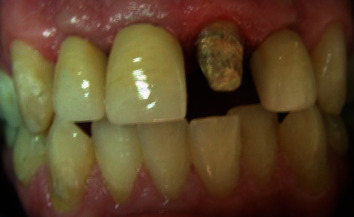
Preparation of cast post.

**Figure 18:**
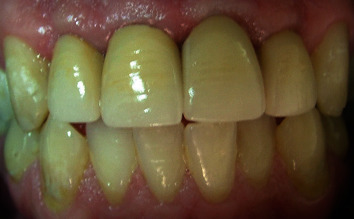
Placement of crowns.

A 30-g force was applied, measured using a Dontrix gauge (13). The elastic thread was changed every seven days until the predetermined extrusion was achieved. The extrusion was completed in two months (Figure 16). Endodontic retreatment was undertaken immediately after the stabilization period (3–4 weeks). A clinical examination showed that the root canal treatments of other incisors were satisfactory enough to carry out the restorative procedure. Crown lengthening was undertaken to restore gingival contours properly. After a healing period of 4 weeks, a ferrule of 2 mm was achieved. Cast post-and-core of the left central and definitive coronal restorations (pressed/metal crowns) were then fabricated for all incisors (Figures 17 and 18).

**Figure 19:**
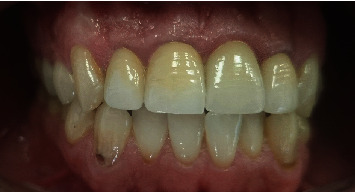
Clinical view after 5 years follow-up.

The patient was followed for five years, and the treatment outcome was favorable (Figure 19).

## 3. Discussion

Extensive caries or crown fractures might create a difficult situation for restoration placement. The significant problem is the lack of adequate coronal ferrule and a compromised biological width. Tooth extrusive movements entail the application of tractional forces throughout the periodontium to induce the marginal apposition of crestal bone [16].

Increased alveolar crest height was achieved through slow orthodontic tooth movements. If the tooth remaining structure is below the alveolar bone and free gingival margin, and if the root length is sufficient to provide support for a coronal restoration, the root can be treated endodontically, followed by orthodontic extrusion. Forced eruption provides more favorable conditions for prosthodontic coronal restorations by guaranteeing proper sealing and esthetics and preserving periodontal tissue health [17]. A histological evaluation by Simon et al. indicated that extrusion of endodontically treated teeth did not pose any apparent problems. They reported that the alveolar process moves in the occlusal direction as the tooth is extruded, followed by bone deposition at the alveolar crest and the interradicular area [18].

The cases reported here consisted of teeth with subgingival carious lesions that were successfully treated using a multidisciplinary approach. Among the treatment options for such cases, such as extraction followed by implant rehabilitation, orthodontic extrusion was deemed the best choice. Implant rehabilitation in such cases often involves surgical procedures to improve the hard and soft tissue profiles of implant recipient sites. Systematic evaluation of interproximal and buccal bone and the amount and type of soft tissue available at the implant site might determine long-term esthetic outcomes. The pink and white esthetic scores (PES and WES) are tools that objectively evaluate implant restorations in the esthetic zone and describe the patient's appraisal of their treatment outcomes [19]. A gingival thickness of ≥2 mm was deemed a thick tissue biotype, and a gingival thickness of <1.5 mm was deemed a thin tissue biotype. The prevalence of thin gingival biotype is 43% in the maxillary incisor area [20, 21]. The PES/WES scores of patients with a thick gingival biotype are significantly higher than those with a thin gingival biotype. A thin gingival biotype might fail to reach the clinically acceptable PES level [22, 23]. Peri-implant soft tissue stability depends on the gingival biotype as a significant parameter for the esthetic outcome of the implant restoration in the esthetic zone [24]. The gingival tissue's potential to cover any underlying material is of utmost importance for achieving aesthetic outcomes, especially in implant, regenerative, and restorative procedures, where subgingival metallic restorations are used [25]. During the restoration of maxillary central incisors, it is important to preserve or reconstruct the interdental papillae. Orthodontic extrusion enhances the quality and quantity of the papilla [26].

The simplified forced eruption technique described has several advantages over other methods; because orthodontic band and bracket are not required, with bonded brackets, there is a necessity to align the anteriors, and time will be lost as a result. Furthermore, reciprocal forces of intrusion might act on the adjacent teeth. In this method, using dentinal pin on the buccal surface of the tooth instead of using a hook inside the root canal has led to overcome the problem of short distance between the hook and the wire to place the elastics; in addition, we will not have occlusal interferences in the palatal surface of the tooth. Therefore, this method can be easily done with the equipment available in any office.

One point that should be considered is that one of the most common ways to prevent caries during orthodontic treatment is to use topical fluoride products such as fluoride varnish and mouthwash. These substances lead to the formation of fluorhydroxyapatite in enamel prisms, which are more resistant to acid attacks and can interfere with the bonding procedures of the composite to the enamel. Therefore, it is recommended that there be more than 15 days delay between the application of topical fluoride products and the bonding procedure to the enamel to achieve the optimal bond strength [27].

Whatever appliance is used, the patient must be seen every 1 to 2 weeks to reduce the occlusal surface of the tooth being extruded, control inflammation, and monitor progress. During the eruptive phase, the application of gel with postbiotics can be useful to prevent gingivitis following plaque accumulation, which can lead to attachment loss and tissue destruction due to the presence of periopathogenic bacteria [28].

The patient's age, the distance the tooth is to be moved, and the PDL viability determine the time required for forced eruption [29]. The extrusion rate used in these cases was similar to that recommended by another author [17]. After two months of extrusion, 3–4 mm of the roots were exposed at an average speed of 0.5 mm/week, while other authors reported an average extrusion rate of 1 mm/week [11, 30]. Slow extrusion requires a 15-g force for the delicate root of a lower incisor tooth, with a 60-g force for a molar tooth. According to some authors, the maximum force for a slow movement should not exceed 30 g [31, 32]; however, rapid extrusion is accomplished by applying forces >50 g. Therefore, extended retention periods are necessary for stabilizing the tooth for the remodeling and adaptation of the periodontal structures to the new tooth position [33]. In these cases, a 30-g force was exerted.

Pressed/metal crowns were used for final restorations. Pre- and postoperative comparisons revealed excellent esthetic results. Moreover, compared to all-ceramic restorations, which require a shoulder finish line, this treatment was considered more conservative. The application of toothpaste and mouthwash containing paraprobiotics-based agents would be an effective protocol for the home maintenance of oral health [34].

## 4. Conclusion

Orthodontic extrusion or forced eruption is a conservative treatment modality to restore fractured or extensively damaged teeth at a subgingival level to preserve the natural tooth and maintain periodontal architecture.

## Data Availability

The datasets used during the treatment are available from the corresponding author on reasonable request.

## Additional Points

*Key Clinical Message*. The forced eruption is an appropriate method for preserving teeth with large destruction of submarginal tissue if case selection was done precisely.
